# Cognitive screening test in primary care: cut points for low education

**DOI:** 10.11606/S1518-8787.2018052000462

**Published:** 2018-11-14

**Authors:** Juliana Emy Yokomizo, Katrin Seeher, Glaucia Martins de Oliveira, Laís dos Santos Vinholi e Silva, Laura Saran, Henry Brodaty, Ivan Aprahamian, Monica Sanches Yassuda, Cássio Machado de Campos Bottino

**Affiliations:** I Universidade de São Paulo. Faculdade de Medicina. Programa Terceira Idade (PROTER), Instituto de Psiquiatria. São Paulo, SP, Brasil; II University of New South Wales. Dementia Collaborative Research Center. Sydney, New South Wales, Australia; III Universidade de São Paulo. Escola de Artes, Ciências e Humanidades. São Paulo, SP, Brasil; IV Departmento de Clínica Médica. Faculdade de Medicina de Jundiaí. Jundiaí, SP, Brasil

**Keywords:** Aged, Cognitive Dysfunction classification, Dementia, diagnosis, Educational Status, Psychometrics, Geriatric Psychiatry, Surveys and Questionnaires, utilization

## Abstract

**OBJECTIVE:**

To establish the diagnostic accuracy of the Brazilian version of the General Practitioner Assessment of Cognition (GPCOG-Br) compared to the Mini-Mental State Examination (MMSE) in individuals with low educational level.

**METHODS:**

Ninety-three patients (≥ 60 years old) from Brazilian primary care units provided sociodemographic, cognitive, and functional data. Receiver operating characteristics, areas under the curve (AUC) and logistic regressions were conducted.

**RESULTS:**

Sixty-eight patients with 0–4 years of education. Cases (n = 44) were older (p *=* 0.006) and performed worse than controls (n *=* 49) on all cognitive or functional measures (p < 0.001). The GPCOG-Br demonstrated similar diagnostic accuracy to the MMSE (AUC = 0.90 and 0.91, respectively) and similar positive and negative predictive values (PPV/NPV, respectively: 0.79/0.86 for GPCOG-Br and 0.79/0.81 for MMSE). Adjusted cut-points displayed high sensitivity (all 86%) and satisfactory specificity (65%–80%). Lower educational level predicted lower cognitive performance.

**CONCLUSIONS:**

The GPCOG-Br is clinically well-suited for use in primary care.

## INTRODUCTION

As life expectancy is growing worldwide, the aging of societies is a global phenomenon. In this context, health services are facing increasing pressure to care for older adults. Age is the most important risk factor for dementia, and projections estimate there will be 135 million people with the disease in 2050 ^1^ , 71% of whom will live in low or middle-income countries.

Misdiagnosis rates for dementia are high in primary care even in high-income countries ^2^ , and early detection of cognitive impairment can improve treatment and reduce costs. Among the 20 million Brazilians over 60 years old, approximately 7% have dementia ^3^ . Undoubtedly, the health system needs to strengthen its means to provide good quality and cost-effective services for this population group.

The General Practitioner Assessment of Cognition (GPCOG) ^4^ was specifically developed as a screening test for primary health care, differentiating it from almost all other assessment instruments, which were created to fulfill demands of the clinical setting. In comparison to other tests, the GPCOG adds a brief cognitive evaluation of the patient and an informant-rated assessment of functional abilities. This strategy makes the instrument time-efficient because it assesses both cognition and everyday function. Also, several reviews have positively recommended the GPCOG based on the time required for its administration (4–6 minutes), as well as its diagnostic accuracy, predictive values and minimal cultural, language and educational bias^5–7^. Besides, the GPCOG is free, and its application requires simple materials (just paper and pen). Reliability is high for the patient section and satisfactory for the informant section. Reported sensitivity rates are 82%–85%, and specificity rates range between 83% and 86% ^7^ . Also, the GPCOG performs at least as well as the Mini-Mental Status Examination (MMSE), while being faster to administer ^5^ ^,^ ^7^ .

The GPCOG has been studied in different languages and sociocultural contexts. It was adapted for French ^8^ , Italian ^9^ , Korean ^10^ , and Chinese ^11^ populations. The psychometric properties observed in the adapted versions were very similar to those in the original publication, and all adaptation studies suggested the same cut-off scores as the original proposal ^4^ . Furthermore, the GPCOG was shown to be better in ruling out dementia in a sample of community dwellers when compared to the Rowland Universal Dementia Assessment Scale and to the MMSE ^12^ .

Cognitive screening for dementia in low and middle-income countries should be culturally and educationally fair ^13^ . Some instruments like the MMSE are biased by the subject’s educational level ^14^ , age, socioeconomic status and symptoms of depression ^15^ . Total scores either need to be adjusted ^15^ or different cut points need to be used in order to correctly classify individuals who are illiterate or have very low educational levels ^16^ . The GPCOG, and particularly its informant component, have been found to be less biased ^5^ ^,^ ^6^ . In any case, individuals with low educational attainment have been excluded from some previous studies ^9^ , and further investigations on sociodemographic biases should be carried out.

In Brazil, approximately one-third of the older adult population is illiterate ^17^ . Some studies have investigated the efficacy of cognitive screening tests in the Brazilian population, but not in a primary care sample ^18^ ^,^ ^19^ (for an exception, see ^20^ ). Therefore, it is crucial to verify whether cognitive screening tools recommended and validated in high-income countries with overall higher educational attainment are as effective for populations with lower educational levels ^7^ .

This study aimed to analyze GPCOG performance in a Brazilian cohort with a sample predominantly comprised of individuals with less than five years of education, by establishing the test’s psychometric properties in comparison to the MMSE. Based on previous studies ^8^ ^,^ ^9^ ^,^ ^11^ , we hypothesized that the GPCOG would be at least as effective as the MMSE in differentiating cases of dementia from controls. We further expected, based on evidence derived from research on the MMSE ^16^ , the Cambridge Cognitive Examination (CAMCOG) ^21^ and the Bayer Activities of Daily Living (BADL) ^22^ , that the optimal cut points in our cohort would be lower than those established for well-educated samples.

## METHODS

### Participants

This prospective study was part of a larger Brazilian validation project for screening instruments ^20^ . Data collection was conducted between June 2012 and February 2014. One hundred and nineteen subjects aged ≥ 60 years from two primary care units (PCU) located in the eastern region of São Paulo participated in the study.

To increase dementia awareness, training sessions were offered to participating staff of the units. Afterwards, community health workers (CHW) were asked to identify consecutive patients who had suspected dementia, or subjective complaints about their memory or any other cognitive domain. The workers were also asked to refer an available informant to participate in the study. Families unable to attend a PCU were assessed at home.

Individuals diagnosed with depression, delirium *,* psychiatric or neurological disorders other than dementia, current or previous alcohol abuse – as defined by a score > 1 on the CAGE scale – and severe visual or auditory deficits were excluded.

### Ethics

All participants provided written informed consent. Information and consent sheets were read aloud for illiterate participants, and verbal witnessed consent was obtained. The project was approved by the Research Ethics Committee of the Municipal Secretariat for Health (CAAE 199.444/11) and by the Research Ethics Committee of the Faculdade de Medicina of the Universidade de São Paulo.

### Instruments

The GPGOG’s original author was contacted for permission to perform the translation. Two neuropsychologists (JEY, MSY) and a psychogeriatrician (CMCB) wrote the first Portuguese draft. Another neuropsychologist, expert in translating cognitive instruments and not directly involved in this study, made fine adjustments for cultural adaptation. All the items of the GPCOG were derived from three sources, mostly the CAMCOG (all 9 items of the patient section) and the BADL (four of six items of the informant section). Both instruments had been previously translated to Portuguese ^21^ ^,^ ^22^ , which ensured sociocultural suitability to our population. Name and address in the recall task were adapted to local nouns. The most up-to-date version was reverse-translated by an independent neuropsychologist fluent in English.

Like in the original version, the GPCOG-Br patient section consists of four cognitive tasks: time orientation (date), visuospatial skills (clock-drawing test), episodic memory (to report a recent news event) and delayed recall (name and address). The informant section is comprised of six questions concerning patients’ current daily function (e.g., remembering things that have happened recently, recalling recent conversations, finding the right words while speaking, managing financial issues and medications, and needing assistance with transportation), as compared to several years ago.

The MMSE is a screening instrument widely used to detect cognitive impairment ^16^ . The CAMCOG ^23^ is a brief neuropsychological battery which assesses the following main areas of cognition: attention, language, memory, visual functions, praxis, and abstraction. The Informant Questionnaire on Cognitive Decline in the Elderly (IQCODE) ^24^ and the B-ADL ^22^ are informant-rated measures for the decline of daily function.

### Procedure

Gerontologists administered the GPCOG-Br and collected patients’ sociodemographic details (verified by informants) and psychiatric history, including alcohol abuse. Neuropsychologists were blinded to the GPCOG-Br results and administered Brazilian versions of the MMSE ^16^ , the CAMCOG ^23^ , the IQCODE ^24^ and the B-ADL ^22^ . Each test session lasted approximately 90 minutes. The results (except in the case of the GPCOG-Br) were later discussed by a team consisting of neuropsychologists (JEY, LSF, MSY) and a psychogeriatrician (CMCB). Cut-off scores from previous national studies were used ^21^ ^,^ ^22^ ^,^ ^25^ . Participants were classified as controls (no cognitive impairment) and cases (cognitive impairment, probable dementia) by a multidisciplinary team of experts, based on DSM-IV criteria. A brief summary report was sent to PCU managers to be included in patients’ clinical records.

### Statistical Analyses

All statistical analyses were performed using SPSS software version 22. Differences between groups were compared using chi-square and Student’s t-test for categorical and continuous variables, respectively. To control for potential confounders, ANCOVA was conducted using sociodemographic variables (significantly different between groups) as covariates, after confirming data were sufficiently normally distributed. Correlations between the GPCOG-Br and other variables were calculated using Pearson’s correlation coefficient.

Receiver Operating Characteristic (ROC) curves and areas under the curve (AUC) were calculated for the GPCOG-Br patient and informant sections, the GPCOG-Br total score and the MMSE, in order to establish diagnostic accuracy. Confidence intervals for AUC were compared to establish whether the diagnostic accuracy was significantly different between tests and scores. Optimal cut points for each tool were chosen so as to maximize both the sensitivity and specificity of the differentiation between cases and controls. Positive and negative predictive values and positive and negative likelihood ratios were also calculated. To determine whether GPCOG-Br and MMSE scores were biased by participants’ sociodemographic characteristics, we conducted exploratory hierarchical linear regressions using age, gender, education and socioeconomic status (SES) as predictors for GPCOG-Br (total, patient and informant) and MMSE scores after controlling for dementia status. The level of significance for all statistical analyses was set to < 0.05 (two-sided).

## RESULTS

Among 119 participants who responded to the protocol, 26 were excluded (alcohol abuse = 8; current depressive disorder = 7; severe auditory or visual impairment = 4; other psychiatric disorder = 2; informant not available = 3; and quit before finishing the protocol = 2). None of the participants reported discomfort during the assessments and none of them requested to withdraw. Table 1 presents the sociodemographic characteristics of the remaining 93 participants, divided into cases (n = 44) and controls (n = 49). Cases were significantly older than controls (p = 0.006), but did not significantly differ on gender and education. Most participants attended school for 1–4 years (45%). However, one-quarter had no formal education; among these, only one case and six controls were able to read and write rudimentarily.

**Table 1:** Sample characteristics of the remaining 93 participants from the eastern region of São Paulo.

Variable	Cases (n = 44)	Controls (n = 49)	Test value	df	p
Age	76.8 (6.9)	72.5 (7.8)	2,790	91	0.006
Female gender – n (%)	36 (81.8%)	40 (81.6%)	0,001	1	1.00
Education – n (%)					
No formal education	12 (27.3%)	12 (24.4%)	11,712	8	0.127
1–4 years	22 (50%)	20 (40.8%)			
5–8 years	5 (11.4%)	10 (20.4%)			
> 8 years	5 (11.4%)	7 (14.3%)			
GPCOG-Br patient score – M (SD)	1.77 (2.27)	5.21 (2.18)	31,940	2	< 0.001
Max. score	9	9			
GPCOG-Br inform. score – M (SD)	1.84 (1.53)	4.10 (1.60)	29,189	2	< 0.001
Max. score	6	6			
GPCOG-Br total score – M (SD)	3.64 (3.35)	9.31 (2.95)	45,595	2	< 0.001
Max. score	15	15			
MMSE – M (SD)	15.2 (4.97)	23.5 (3.89)	49,902	2	< 0.001
Max. score	24	30			
CAMCOG – M (SD)	46.48 (17.06)	77.06 (11.68)	71,058	2	< 0.001
Max. score	81	102			
IQCODE – M (SD)	3.87 (0.64)	3.22 (0.23)	25,681	2	< 0.001
Max. score	5.73	3.65			
B-ADL – M (SD)	5.82 (2.50)	2.62 (1.38)	32,369	2	< 0.001
Max. score	10	6			

df: degrees of freedom; n: sub-sample; M: mean; SD: standard deviation; GPCOG-Br: General Practitioner Assessment of Cognition – Brazilian version; MMSE: Mini-Mental Status Examination; CAMCOG: Cambridge Cognitive Examination; IQCODE: Informant Questionnaire on Cognitive Decline in the Elderly; B-ADL: Bayer Activities of Daily Living

Results of all cognitive and functional tests were clearly distinct between groups (p < 0.001), even after statistically controlling for age differences between cases and controls. Cases performed worse than controls on both GPCOG-Br sections and on the GPCOG-Br total score. Their MMSE and CAMGOG scores were 8 and 20 points lower, respectively, indicating that cases had more functional impairment ( Table 1 ).

Sensitivity, specificity, positive predictive value (PPV), negative predictive value (NPV) and AUC of the GPCOG-Br total score, patient and informant sections and the MMSE, using the original as well as the cut points modified for cultural and educational fairness, are shown in Table 2 . ROC curves were similar ( Figure 1 ). Despite the GPGOC-Br’s brevity, it had a similar diagnostic accuracy to the MMSE (AUC = 0.90 and 0.91, respectively), as indicated by the largely overlapping confidence intervals. Applying the original cut points, sensitivity was very high for the GPCOG-Br patient and informant sections and for total score, and for the MMSE, indicating that between 95% and 97% of cases were correctly classified as impaired. On the other hand, specificity was very low, correctly classifying 16%–45% of controls as healthy. The adjusted cut points showed good sensitivity and specificity for all instruments, and moderate specificity for the GPCOG-Br informant section (65%). The GPCOG-Br total score was as good as the MMSE in ruling out dementia (NPV = 0.86 and 0.81, respectively). Misclassification rates ranged from 17% for the GPCOG-Br total score to 25% for the GPCOG-Br informant section.

**Table 2:** Psychometric properties for GPCOG-Br patient and informant sections, GPCOG-Br total score and MMSE, with comparison between the proposed and the original cut-off points.

Variable	GPCOG-Br Patient Section Proposed Original		GPCOG-Br Informant Section Proposed Original		GPCOG-Br Total Score Proposed Original		MMSE Total Score Proposed Original	
Cut-off point	3/4	7/8	3/4	4/5	6/7	10/11	19/20	24/25
Maximum score	9		6		15		30	
N	93	93	93	93	93	93	93	93
Sensitivity (%)	86	95	86	95	86	95	79	97
Specificity (%)	75	16	65	43	80	33	81	45
PPV	0.76	0.50	0.69	0.60	0.79	0.56	0.79	0.61
NPV	0.86	0.80	0.84	0.91	0.86	0.89	0.81	0.95
AUC	0.873		0.841		0.896		0.905	
AUC 95%CI	0.795–0.951		0.759–0.922		0.827–0.964		0.846–0.965	
AUC SE	0.040		0.042		0.035		0.030	
Misclassification (%)	19	46	25	32	17	38	19	30

N: sample; PPV: positive predictive value; NPV: negative predictive value; AUC: area under the curve; SE: standard error; GPCOG-Br: General Practitioner Assessment of Cognition – Brazilian version; MMSE: Mini-Mental Status Examination

**Figure:**
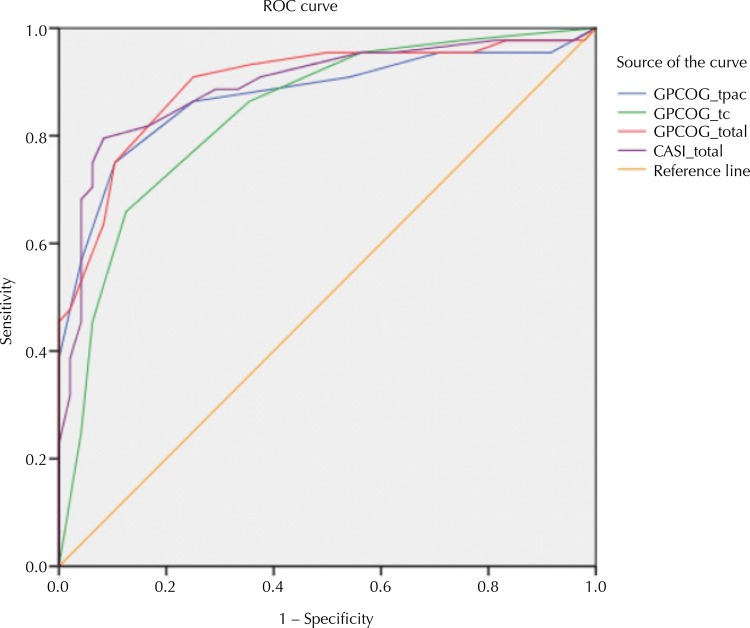
Receiver Operating Characteristics (ROC) curves for the GPCOG-Br and the MMSE, total sample. (n = 93)

GPCOGBr_patient: General Practitioner Assessment of Cognition – Brazilian Version, patient section; GPCOGBr_informant: General Practitioner Assessment of Cognition – Brazilian Version, informant section; GPCOGBr_total: General Practitioner Assessment of Cognition – Brazilian Version, total section; MMSE: Mini-Mental Status Examination

When comparing ROC in participants with less than five years of education (n *=* 66) and participants with five years or more (n = 27), areas under the curve (AUC) were not significantly different. (Standardized differences for: GPCOG-Br total = 0.119 [SE = 0.1], p = 0.24; GPCOG-Br patient = 0.153 [SE = 0.11], p = 0.16; GPCOG-Br informant = -0.061 [SE = 0.08], p = 0.44; MMSE = 0.034 [SE = 0.08], p = 0.68).

Alternative GPCOG-Br cut-off scores are shown in Table 3 . Respective sensitivity, specificity rates and likelihood ratios (LR) were included. Small effects were found for positive likelihood ratios in almost all the optimized cut-off scores. Moderate positive LR was found when the two-thirds cut-off score for the GPCOG-Br patient section was used. Negative LR presented moderate effect on all the selected cut points.

**Table 3:** Alternative cut-off points with respective sensitivity and specificity rates and positive and negative likelihood ratio, for GPCOG-Br sections and total score.

Variable	Cut-off point	Sensitivity	Specificity	PLR	NLR
GPCOG-Br patient section	2/3	0.75	0.90	7.35	0.27
	3/4	0.86	0.75	3.52	0.18
	4/5	0.89	0.60	1.14	0.27
GPCOG-Br informant section	2/3	0.66	0.87	2.49	0.20
	3/4	0.86	0.65	1.67	0.10
	4/5	0.95	0.44	4.23	0.17
GPCOG-Br total score	5/6	0.75	0.90	1.41	0.14
	6/7	0.86	0.79	4.33	0.25
	7/8	0.91	0.75	1.77	0.05

PLR: positive likelihood ratio; NLR: negative likelihood ratio; GPCOG-Br: General Practitioner Assessment of Cognition – Brazilian version

**Table 4:** Sociodemographic predictors of GPCOG-Br and MMSE scores after controlling for participants’ diagnostic status.

Variable	GPCOG-Br patient		GPCOG-Br informant			GPCOG-Br total		MMSE	
			
	Model 1	Model 2	Model 1	Model 2	Model 3	Model 1	Model 2	Model 1	Model 2
Group/Diagnosis	0.613^c^	0.544^c^	0.059^c^	0.520^c^	0.523^c^	0.673^c^	0.596^c^	0.688^c^	0.607^c^
Gender		-0.151^a^		0.048	0.051		-0.083		-0.146^a^
Age		-0.115		-0.187^a^	-0.188^a^		-0.157^a^		-0.176^a^
Education		0.385^c^		0.168	0.170		0.332^c^		0.308^c^
SES		0.077		0.061	0.058		0.081		0.135^a^
Informant education					-0.018				
Adjusted R^2^	0.37^c^	0.55^c^	0.34^c^	0.39^b^	0.38^n.s.^	0.45^c^	0.59^c^	0.47^c^	0.62^c^

SES: socioeconomic status; GPCOG: General Practitioner Assessment of Cognition – Brazilian Version; MMSE: Mini-Mental Status Examination

Standardised regression coefficients (i.e. betas) are displayed with corresponding p-value: ^a^ p < 0.05; ^b^ p < 0.01; ^c^ p < 0.001; ^n.s^: not significant.

## DISCUSSION

This is the first validation study of a cognitive screening instrument specifically designed for the primary care context in Brazil. Despite its brevity, the GPCOG-Br assesses both cognition and function, which is particularly advantageous for evaluating patients with low educational level.

Our data indicate that the GPCOG was easily translated and adapted to Portuguese (GPCOG-Br). Its administration is easy and intuitive, so routine training could be offered to community health workers at minimal costs. In some developing countries, community health workers play a key role in primary care. A study conducted in São Paulo found that a 3-hour psychoeducational program for these workers improved their detection of possible dementia cases after only two weeks ^26^ . Training in the operation of screening instruments like the GPCOG-Br could further improve the detection of dementia. Individuals who screen positive on the GPCOG-Br could then be streamlined for further testing and professional management.

Data from AUC presented here are very similar to data reported in the original article ^4^ , showing that the GPCOG-Br is an efficient instrument for identifying cases requiring further streamlined testing and management. The GPCOG-Br total score with adjusted cut points correctly classified 83% of the participants, performing very similarly to the MMSE (81%). For every 100 individuals who screened positive on the GPCOG-Br total or MMSE using our adjusted cut points, 79 actually met DSM-IV criteria for dementia (PPV). Besides, compared to the MMSE (NPV), the GPCOG-Br total score correctly identified additional five non-impaired cases for every 100 cases screened negative.

Similar to any screening instrument, adoption of the GPCOG-Br can entail a major discussion regarding sensitivity and specificity. We found a relatively low level of specificity at an ideal level of sensitivity. Further item-by-item analyzes could verify if there are one or more items able to contribute to diagnosis prediction or if there are one or more items that act as diagnosis confounders. An alternative could be to include some extra cognitive task, and check if this boosts specificity. A Brazilian study ^27^ added a visuoconstructional task to the Mini-Cog, but found that the inclusion did not improve the performance of the test, and concluded that this may have occurred because the task demanded certain executive functions that are mainly developed in formal education.

In any case, we believe that the GPCOG adaptation presented in this study could still be used in a primary care setting. In this context, screening for dementia needs to identify all possible cases, which is more relevant than arriving at a definite diagnosis. The diagnosis itself can be obtained in subsequent steps, after further evaluation.

The dementia prevalence in our sample was high, and some subjects presented moderate symptoms (e.g., CAMCOG standard deviation was > 17). This scenario might be common in developing countries, where dementia is still a hidden problem, since there is a lack of information to help people in the community detect signs and differentiate dementia from normal aging ^28^ .

In many aspects, our sample was very distinct from the other studies’ samples. The French study was comprised of patients of a psychogeriatric service with at least one psychiatric diagnosis other than dementia ^8^ , whereas we excluded individuals with current psychiatric comorbidities. The Italian study ^9^ did not include individuals with low educational level or comorbidities such as diabetes, which frequently occurred in our sample. In addition, the minimal age was 5 to 10 years lower in both the Italian and Chinese cohorts ^9^ ^,^ ^11^ .

Our sample is similar to other Brazilian cohorts with respect to sociodemographic characteristics such as rural background, poor nutritional conditions during development, low educational level and reduced opportunities during adulthood ^29^ . These factors may introduce bias to cognitive screening instruments. The MMSE, for instance, performed poorly in a sample of illiterate Brazilians in a population-based study (n *=* 1,933), even with adjusted cut points, deducting three points from the total score compared to individuals who attended school for one or more years ^14^ .

The effects of formal education on cognition seem to be complex. A Mexican study ^29^ assessed the performance of 806 subjects (aged 16–85 years, educational level 0–10 years) via a neuropsychological battery. Individuals with lower education showed significantly worse performance in almost all tests. Groups with higher educational levels presented a lower decline on tasks such as word recall across all age ranges. Education predicted > 20% of the variance for tests demanding constructional and conceptual abilities. Word recall and constructional praxis are very commonly used in screening tests. The authors argued that low education should be taken into account when diagnosing dementia.

Our study had some limitations, such as a smaller sample size compared to previous GPCOG validation studies. Further researches should use a sample size formula to potentiate statistical analyzes. We recruited consecutive PCU patients, considered in need of cognitive assessment by community health workers, rather than screening all PCU patients over a certain age. Diagnostic decisions were made only using cognitive assessment and the provided clinical information – other information such as blood tests and neuroimaging was not available. We did not assess the severity of dementia, which could clarify the frequency of mild cases with higher chances of being misclassified even by experts. A larger sample size could both control the severity of dementia and allow further analyzes in order to check if educational level is a predictor for the diagnosis. While other studies did not find educational biases, this may have been because their samples were mostly comprised of highly educated subjects.

Despite the fact that the number of subjects discharged due to the exclusion criteria was small (n = 26), the criteria may have affected the representativeness of the final sample. Further analyses involving these subjects could not be performed because many of the excluded participants (e.g., subjects with severe hearing impairment) did not finish the assessment.

In summary, our main findings are new cut points for individuals with low educational level. The previous GPCOG adaptations ^8^ ^,^ ^9^ ^,^ ^11^ presented the same cut points as the original study ^4^ . Sample characteristics may partially explain the different cut points found here. Socioeconomic disadvantage in early life might be associated with a higher prevalence of dementia, possibly because growing up in poor areas usually leads to poor nutrition, lower education level, low-skilled jobs offer and, from a developmental perspective, a reduced cognitive reserve ^30^ . Further studies should involve larger samples with different socioeconomic and educational backgrounds, to clarify these hypotheses.
